# The radiographic, pulmonary, and clinical outcomes of patients with severe rigid spinal deformities treated via halo‐pelvic traction

**DOI:** 10.1186/s12891-021-03953-y

**Published:** 2021-01-23

**Authors:** Jian Chen, Wen-yuan Sui, Jing-fan Yang, Yao-long Deng, Jing Xu, Zi-fang Huang, Jun-lin Yang

**Affiliations:** 1 Spine Surgery Center, Xinhua Hospital, Shanghai Jiaotong University School of Medicine, 1665 Kongjiang Road, Shanghai, China; 2 Department of Orthopaedic Surgery, the 1st Affiliated Hospital of Sun Yat-sen University, Guangzhou, Guangdong China

**Keywords:** Severe rigid spinal deformity, Halo‐pelvic traction, Halo‐gravity traction, Pulmonary function

## Abstract

**Background:**

The severe rigid deformity patients with pulmonary dysfunction could not tolerate complicated corrective surgery. Preoperative traction are used to reduce the curve magnitude and improve the pulmonary function before surgery, including halo-gravity traction (HGT) and halo-pelvic traction (HPT). The present study aimed to retrospectively compare the radiographic, pulmonary and clinical outcomes of preoperative HGT and HPT in severe rigid spinal deformity with respiratory dysfunction.

**Methods:**

81 cases of severe rigid kyphoscoliosis treated with preoperative traction prior to corrective surgery for spinal deformity between 2016 and 2019 were retrospectively reviewed. Two patient groups were compared, HPT group (*N* = 30) and HGT group (*N* = 51). Patient demographics, coronal and sagittal Cobb angles and correction rates, pulmonary function, traction time, osteotomy grade, and postoperative neurological complications were recorded for all cases.

**Results:**

The coronal Cobb angle was corrected from 140.67 ± 2.63 to a mean of 120.17 ± 2.93° in the HGT group, and from 132.32 ± 4.96 to 87.59 ± 3.01° in the HPT group (mean corrections 15.33 ± 1.53 vs. 34.86 ± 3.11 %) (*P* = 0.001). The mean major sagittal curve decreased from 134.28 ± 3.77 to 113.03 ± 4.57° in the HGT group and from 129.60 ± 8.45 to 65.61 ± 7.86° in the HPT group (*P* < 0.001); the mean percentage corrections were 16.50 ± 2.13 and 44.09 ± 9.78 % (*P* < 0.001). A significant difference in the pulmonary function test results was apparent between the two groups; the mean improvements in the FVC% of the HGT and HPT groups were 6.76 ± 1.85 and 15.6 ± 3.47 % (*P* = 0.024). The HPT group tended to exhibit more FEV% improvement than the HGT group, but the difference was not significant (5.15 ± 2.27 vs. 11.76 ± 2.22 %, *P* = 0.91).

**Conclusions:**

Patients with severe rigid kyphoscoliosis who underwent preoperative HPT exhibited better radiographic correction of the deformity, and pulmonary function, and required fewer osteotomies compared to the HGT group. Thus, HPT may be useful for severe rigid spinal deformity patients with pulmonary dysfunction.

## Background

Surgical correction of severe spinal rigid deformity is challenging and is associated with substantial risks of mortality and morbidity, neurological injury, and even permanent paralysis. The high incidence of pulmonary dysfunction in severe rigid deformity patients is the unavoidable problem for the surgeon during the perioperative period [1, 2]. Pulmonary dysplasia in such patients is attributable principally to the low thoracic volume; volumes during both inspiration and expiration are lower than those of normal subjects [3]. The severity of respiratory impairment is closely linked to the extents of the curves, especially the thoracic and thoracolumbar curves [4, 5]. Irreversible pulmonary function damage, and even respiratory failure, can develop in patients with extremely severe scoliosis [6]. It is challenging to perform corrective surgery for extremely severe scoliosis patients, since their preoperative pulmonary dysfunction could not tolerate such complicated and long time surgery [7–9]. Furthermore, large-angle orthopedics significantly increase the risk of postoperative neurological complications [10].

To reduce such risks, surgeons are encouraged to use any possible means to reduce the major curve magnitude and improve the pulmonary function prior to corrective surgery; the methods include halo-gravity traction (HGT), halo-femoral traction (HFT), and halo-pelvic traction (HPT) [11–13]. Preoperative traction can reduce the main curvature, decreasing the difficulty of operation and the risk of neurological complications [14]. During traction, pulmonary function may improve remarkably because of the reduction in curve magnitude accompanied by careful respiratory training.

HGT assists the curing of severe, pediatric spinal deformities, with no apparent postoperative complications [14]. HGT features the imposition of a low traction force with no need for bedrest, and improves coronal- and sagittal-plane deformities. HGT is well-tolerated, especially by pediatric patients. Most subjects in earlier studies were pediatric patients. HGT decreases the probability of aggressive osteotomy and prevents neurological complication in corrective operation. Sponseller et al. reported that patients treated via HGT were less likely to require vertebral column resection [15]. HFT after spinal release, combined with second-stage posterior correction, has also been proposed to treat severe kyphoscoliosis [16]. However, halo gravity traction has poor strength, low efficiency and is not suitable for the patients with rigid deformity. HFT requires absolute bedrest and contribute to joint stiffness, which was not suitable for long-term preoperative traction. Therefore, HPT has become the most commonly used traction technology for severe rigid scoliosis at present due to its powerful distraction forces.

However, few attention has been paid to the benefit of HPT in improving preoperative clinical conditions, such as curve rigidity and pulmonary function. For ensuring the clinical efficacy of patients, it is essential to optimize preoperative clinical conditions prior to the corrective surgery. In our center, HGT was used for preoperative traction in the early year, but we found that the traction effect of some patients was not ideal, including poor improvement of lung function and nutritional status due to the “plateau effect”. So we chose HPT as the preoperative traction method in recent years. This study aimed to retrospectively compare the therapeutic outcomes of preoperative HGT and HPT prescribed for patients with severe rigid spinal deformities (Cobb angle > 100°).

## Methods

### Study design and ethics approval

This work was approved by our institutional review board. Informed consent was acquired from all patients and their relatives included in the study. We retrospectively reviewed patients with severe, rigid kyphoscoliosis (Cobb angle > 100°; N = 81) treated via traction prior to corrective surgery between 2016 and 2019. Two patient groups were compared, the preoperative halo-gravity traction (HGT) group (N = 51) and the preoperative halo-pelvic traction (HPT) group (N = 30). Patient demographics, coronal and sagittal Cobb angles, correction rates, pulmonary-function data, traction times, osteotomy grades, and postoperative neurological complications were recorded. The inclusion criteria were age > 10 years; a main thoracic scoliosis curve > 100, or kyphosis > 100° when standing (evaluated via whole-spine X-ray imaging) and > 70° on bending sideways; and more than 2 years of follow-up. Patients with any history of spinal surgery were excluded.

### HGT Protocol

**Fig. 1:**
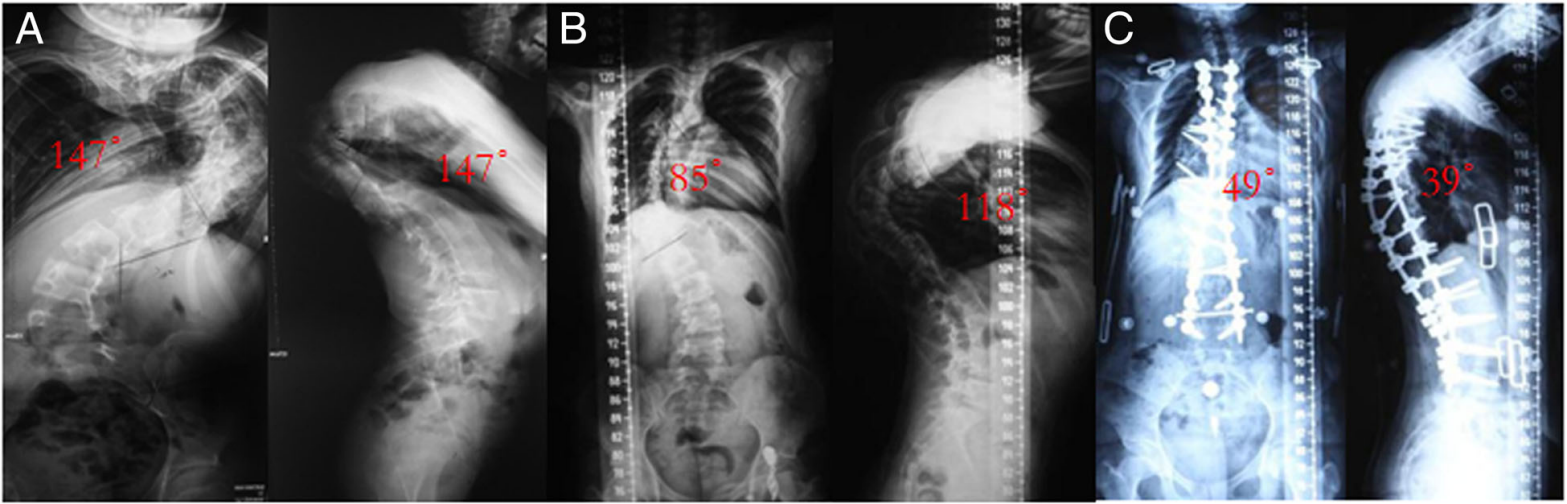
Preoperative application of HGT in a 15-year-old girl with a severe rigid spinal deformity. **a** The patient’s preoperative scoliosis angle(147°) and kyphosis angle(147°). **b** The patient’s scoliosis angle (85°) and kyphosis angle (118°) after 6 months of traction. **c** He was treated with a long posterior reconstruction from T2 to L4 with one-level grade 4 osteotomies at T8/9

Our HGT protocol has been described previously [17]. In brief, the halo frame was placed immediately above the right skull equator and fastened using four pins tightened to 6–8 in-lb. of torque. The initial traction was 20 % of body weight. This was increased to 50 % of body weight over 4 weeks, thus by an average of 10 % per week. Patients were required to maintain traction at all times, except when dining and engaged in personal hygiene. The surgeon checked daily for pin-site infections and neurological signs. Diluted povidone-iodine solution was applied to the shaved epicranial areas around the pin sites at regular intervals. Traction was sustained during surgery (Fig. 1).

### HPT Protocol

**Fig. 2:**
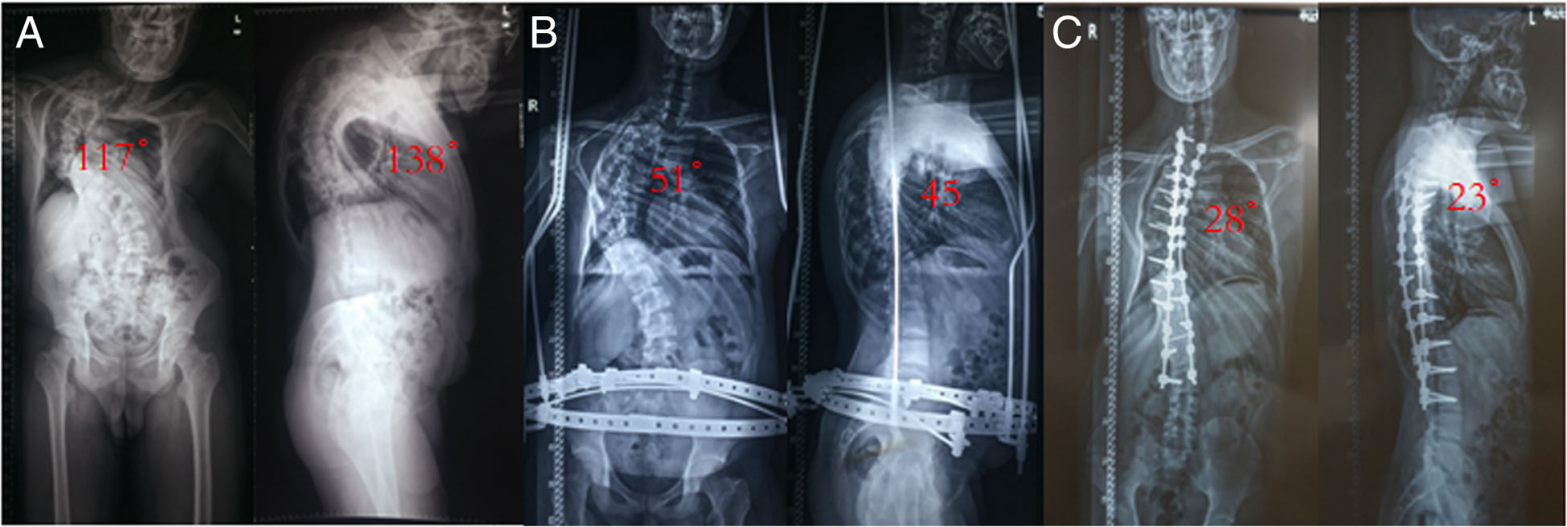
Preoperative application of HPT in a 20-year-old man with a severe rigid spinal deformity. **a** The patient’s preoperative scoliosis angle(117°), kyphosis angle(138°) and general appearance. **b** The patient’s scoliosis angle (51°), kyphosis angle (45°) and general appearance after 4 months of traction. **c** He was treated with a long posterior reconstruction from T1 to L3 with one-level grade 4 osteotomies at T7/8

The HPT apparatus was designed by one of our senior surgeons (YJF). The device features halo and pelvic rings fastened to the cranial and iliac bones respectively, and four correcting rods that align the two rings to allow spinal traction. Local anesthesia is essential during fitting. The cranial bone pins were placed as described above. Two pelvic ring pins were placed on the iliac crest and the posterior superior iliac spine. As shown in Fig. 1, the lengths of the rods connecting the halo to the pelvic rings were shortened by 3–5 mm/day, to distract the spine. The extent of correction, pulmonary function, and nutritional status were evaluated by multidisciplinary consultation to determine whether the patient could tolerate surgery (Fig. 2).

### Radiographic and clinical evaluation

The coronal and sagittal Cobb angles were analyzed preoperatively, after traction, and at 2 weeks after surgery. Preoperative bending radiography was used to assess spinal curve flexibility. Radiographic evaluations were carried out by a senior doctor (SWY). The percentage correction afforded by traction was: (the pre-HGT Cobb angle minus the post-HGT Cobb angle)/the pre-HGT Cobb angle.

### Pulmonary function tests (PFTs)

PFTs were performed before and after traction. The predicted forced vital capacity (FVC%) and forced expiratory volume over 1 s (FEV1 %) were compared pre- and post-traction, as were the relative improvements (post-traction FVC% or FEV% minus pre-traction FVC% or FEV%). Before the correction operation, PFTs will be carried out to determine whether it meets the operation requirements.

### Statistical analysis

Categorical variables were compared using the Pearson chi-squared test and continuous variables via the independent samples *t*-test and variance analysis. The Wilcoxon signed-rank test was employed to compare osteotomy grades. All analyses were performed with the aid of SPSS ver. 19.0 (SPSS Inc., Chicago, IL, USA). A P-value < 0.05 was considered to reflect significance.

## Results

### Patient demographics and clinicopathological data

We retrospectively compared two matched groups, thus a preoperative HGT group (*N* = 51) and an HPT group (*N* = 30). Demographic and clinicopathological data recorded at the initial visits are summarized in Table 1. The groups did not differ in terms of age or sex (both *P* > 0.05). HPT patients underwent less traction (138.30 ± 28.83 days) than did HGT patients (236.18 ± 34.24 days) (*P* = 0.032). As shown in Table 1, the etiological diagnoses of the cases were idiopathic scoliosis (IS, 25 cases), congenital scoliosis (CS, 4 cases), neuromuscular scoliosis (NM, 13 cases), neurofibromatosis (NF, 8 cases), tuberculosis (TB, 1 case) in HGT group, which there were 13 cases of IS, 11 cases of CS, 2 cases of NF, and 4 cases of Marfan syndrome (MF) in HPT group (Table 1).

**Table 1:** The demographic in the HGT group and the HPT group

	HGT(*n* = 51)	HPT(*n* = 30)	p
Age	19.06 ± 0.85	20.0 ± 1.05	0.494
Male/Female	18/33	10/20	0.858
Traction Time	236.18 ± 34.24	138.30 ± 28.83	0.032
Diagnosis	IS (25), CS (4), NM(13), NF (8), TB (1)	IS (13), CS (11), NF (2), MF (4)	-

Abbreviations: *HGT* halo-gravity traction, *HPT* halo-pelvic traction, *IS* idiopathic scoliosis, *CS* congenital scoliosis, *NM* neuromuscular scoliosis, *NF* neurofibromatosis, *TB* tuberculosis, *MF* Marfan syndrome.

### Radiographic evaluation

The mean, preoperative Cobb angle did not differ between the two groups, being 140.67 ± 2.63° in the HGT group and 132.32 ± 4.96° in the HPT group. At the end of traction, immediately preceding fusion, the coronal Cobb angle was corrected to a mean of 120.17 ± 2.93° in the HGT group and 87.59 ± 3.01° in the HPT group (correction mean 15.33 ± 1.53 vs. 34.86 ± 3.11 %, *P* < 0.001). After surgery, the mean major coronal curve decreased to 63.86 ± 2.16° in the HGT group and 44.43 ± 3.43° in the HPT group (*P* < 0.001); the mean percentage corrections were 54.10 ± 1.61 and 67.01 ± 3.04 % (*P* = 0.001). The preoperative sagittal Cobb angle did not differ between the HGT and HPT groups (134.28 ± 3.77 vs. 129.60 ± 8.45°, *P* = 0.569). After traction, the mean major sagittal curve decreased to 113.03 ± 4.57° in the HGT group and to 65.61 ± 7.86° in the HPT group (*P* < 0.001); the mean percentage corrections were 16.50 ± 2.13 and 44.09 ± 9.78 % (*P* < 0.001). After surgery, the coronal Cobb angle was corrected to a mean of 59.14 ± 2.57° in the HGT group and 31.30 ± 3.16° in the HPT group (correction means 55.54 ± 1.76 vs. 71.83 ± 3.84 %) (*P* = 0.001). The radiographic findings are compared in Table 2.

**Table 2:** Radiographic parameters description of the severe scoliotic patients

	HGT(*n* = 51)	HPT(*n* = 30	p
Coronal Cobb angle(°)			0.012
Pre-traction	140.67 ± 2.63	132.32 ± 4.96	0.106
Post-traction	120.17 ± 2.93	87.59 ± 3.01	<0.001
Postsurgery	63.86 ± 2.16	44.43 ± 3.43	<0.001
Traction correction	15.33 ± 1.53	34.86 ± 3.11	<0.001
Postsurgery correction	54.10 ± 1.61	67.01 ± 3.04	0.001
Sagittal Cobb angle(°)			
Pre-traction	134.28 ± 3.77	129.60 ± 8.45	0.569
Post-traction	113.03 ± 4.57	65.61 ± 7.86	<0.001
Postsurgery	59.14 ± 2.57	31.30 ± 3.16	<0.001
Traction correction	16.50 ± 2.13	44.09 ± 9.78	<0.001
Postsurgery correction	55.54 ± 1.76	71.83 ± 3.84	<0.001

Abbreviations: *HGT* halo-gravity traction, *HPT* halo-pelvic traction

### Pulmonary function

The mean preoperative pulmonary function parameters did not differ in terms of the FVC% (43.94 ± 2.59 vs. 46.44 ± 7.22, *P* > 0.05) or the FEV% (40.64 ± 2.38 vs. 49.08 ± 5.46 %, *P* > 0.05) between the HGT and HPT groups. At the end of traction, the FVC% was corrected to a mean of 50.70 ± 2.52 % in the HGT group and to 62.56 ± 6.69 % in the HPT group (*P* = 0.049). The mean FVC% improvement and correction rate differed significantly between the HGT and HPT groups (6.76 ± 1.85 vs. 15.6 ± 3.47 %, *P* = 0.024; 20.52 ± 5.32 % vs. 52.18 ± 21.31 %, *P* = 0.042). Similarly, the mean post-traction FEV% differed significantly between the two groups (46.17 ± 2.43 vs. 59.73 ± 5.25 %, *P* = 0.011). The HPT group appeared to exhibit more FEV% improvement and correction rate compared to the HGT group, but the difference was not significant (*P* > 0.05). The pulmonary function data are compared in Table 3.

**Table 3:** Pulmonary Function Testing Results for Patients Before and After traction

	HGT(*n* = 51)	HPT(*n* = 30)	p
FVC%			
Pre-traction	43.94 ± 2.59	46.44 ± 7.22	0.684
Post-traction	50.70 ± 2.52	62.56 ± 6.69	0.049
Improvement	6.76 ± 1.85	15.6 ± 3.47	0.024
Correction rate	20.52 ± 5.32	52.18 ± 21.31	0.042
FEV%			
Pre-traction	40.64 ± 2.38	49.08 ± 5.46	0.105
Post-traction	46.17 ± 2.43	59.73 ± 5.25	0.011
Improvement	5.15 ± 2.27	11.76 ± 2.22	0.091
Correction rate	12.16 ± 6.29	25.37 ± 6.00	0.222

Abbreviations: *HGT* halo-gravity traction, *HPT* halo-pelvic traction, *FVC%* predicted forced vital capacity, *FEV%* predicted forced expiratory volume over 1 s

### Neurological complications

As showed in Table 4, the HGT group tended to undergo high-grade osteotomies: osteotomy of grade 5 or above accounted for 70.6 % of all cases in the HGT group, while it only accounted for 40 % of those in the HPT group. The neurological complication rates did not differ between the two groups (*P* > 0.05).

**Table 4:** Neurological complication in the HGT group and the HPT group

	HGT(*n* = 51)	HPT(*n* = 30)	p
Osteotomy grade			
6	12	4	0.035
5	24	8	
4	8	13	
3	3	3	
2	4	2	
Neurological complication	12/51	5/30	0.464

Abbreviations: *HGT* halo-gravity traction, *HPT* halo-pelvic traction

## Discussion

In general, patients with severe spinal deformities may suffer from related or independent conditions including restrictive pulmonary diseases, cardiovascular disease, and/or malnutrition. Patients who have not received scoliosis treatment have higher mortality compared with normal persons and the leading causes of death were respiratory failure and cardiovascular disease [18]. The detrimental effect of spinal deformity on pulmonary function is attributable to restriction of lung volume resulted from thoracic dysplasia. Patients with severe spinal deformities exhibited much smaller inspiratory and expiratory thoracic volumes than did controls [3]. Also, a positive correlation was evident between the extent of respiratory disturbance and the severity of scoliosis, particularly thoracic and thoracolumbar scoliosis [4, 5]. Kim found that the Cobb angle was negatively correlated with the FVC% [19]. Aggravation of scoliosis would intensify respiratory impairment, leading to pulmonary hypertension and pulmonary heart disease in case of small airways distortion [20]. Technical advances in vertebral osteotomies and pedicle screw fixation systems have greatly aided the correction of severe kyphoscoliosis, in turn improving long-term pulmonary function [21, 22]. However, posterior orthopedic techniques often need to be combined with thoracoplasty to correct the “razorback” deformity, which destroys thoracic integrity and stability, and affects respiratory function for in a period of time after surgery [23, 24]. Thus, satisfactory preoperative pulmonary function is particularly important in such patients.

Patients with severe, restrictive pulmonary dysfunction exhibit a higher incidence of postoperative pulmonary complications, associated with extubation difficulties and a need for long-term postoperative care [25]. Surgeons and anesthetists find it challenging to operate on patients with extremely severe scoliosis and pulmonary dysfunction. A prime objective when treating a severe spinal deformity is the attainment of a correction that is safe and reproducible. Preoperative traction is a safe means of progressive deformity correction [11–13]. Preoperative traction can reduce the main curvature, leading to the reduction of the operation difficulty and the occurrence of neurological complications [14]. During traction, pulmonary function improves because the scoliosis weakens and respiratory exercise is scheduled, reducing the rate of postoperative pulmonary complications [26].

HGT improves coronal or sagittal deformities via application of relatively low traction forces with no need for bedrest, and is particularly well tolerated by pediatric patients. HGT has been extensively studied in the context of severe spinal deformities. In a case study that included 18 adults with severe kyphoscoliosis, Lenke et al. reported that the major coronal and sagittal Cobb angle were decreased by 18.4 % (14.7°) and 16.8 % (18.8°) after HGT, and 54.7 % (50.4°) and 44.2 % (49.4°) after final surgery [27]. Qiu et al. reported that the Cobb angle averaged 131.21° and was reduced to 107.68° after HGT for 21 adults with severe scoliosis; the correction rate was 17.93 % [28]. However, the traction effect of HGT can reach a plateau in a short time (within 2 weeks) due to its poor strength (up to one-half body weight) and low efficiency, which is not suitable for the severe rigid deformity [29]. HPT was proven to be an ideal therapy for severe and rigid spinal deformity represented by healed tuberculous kyphosis [30]. In the present study, the coronal Cobb angle was corrected from 140.67 ± 2.63 to a mean of 120.17 ± 2.93° in the HGT group, and from 132.32 ± 4.96 to 87.59 ± 3.01° in the HPT group (correction means 15.33 ± 1.53 vs. 34.86 ± 3.11 %) (*P* = 0.001). The mean major sagittal curve decreased from 134.28 ± 3.77 to 113.03 ± 4.57° in the HGT group, and from 129.60 ± 8.45 to 65.61 ± 7.86° in the HPT group (*p* < 0.001); the mean percentage corrections were 16.50 ± 2.13 and 44.09 ± 9.78 % (*P* < 0.001). The coronal Cobb angle was significantly better-corrected in the HPT than the HGT group; patients with severe rigid kyphoscoliosis benefited more from HPT than HGT.

High-level osteotomies are often required when treating severe rigid spinal deformities. HGT helps to lower the grade of osteotomy and reduces the risk of neurological complications after definitive corrective surgery. Sponseller et al. found that patients with HGT were less likely to require vertebral column resection during correction of severe spinal deformities [15]. However, the traction efficiency of HGT in the context of extremely rigid scoliosis remains limited [17]. To the best of our knowledge, the osteotomy grades required after HPT of severely kyphoscoliostic patients have not been previously evaluated. In the present study, the HGT group tended to require more high-grade osteotomies than did the HPT group; osteotomies of grade ≥ 5 accounted for 70.6 % of all osteotomies in the HGT group, but only 40 % of those in the HPT group. These outcomes may reflect the better improvement in the kyphoscoliosis angle of the HPT group than that of the HGT group. A lower osteotomy grade simplifies the operation; and reduces the operation time, intraoperative bleeding, the risk of postoperative complications, the hospital stay, and the need for perioperative rehabilitation.

Deterioration in pulmonary function prior to surgery increases the incidence of postoperative pulmonary complications [31]. As mentioned above, severe scoliotic and kyphotic deformities negatively impact the thoracic cage, disturbing skeletal, muscular, and diaphragmatic function; and reducing respiratory system compliance [32]. Poor respiratory function, not the size of the curve, is the principal cause of physical problems and mortality [33]. When treating a severe thoracic deformity in a patient with a limited thoracic lung volume, it is essential to increase the chest volume, thus improving pulmonary function [33, 34]. However, many studies on the natural courses of pulmonary impairment and severe scoliosis have reported no spontaneous resolution of lung impairment, rather slight drops in the PFT results over time [35], particularly in patients with Cobb angles > 100° and/or FVC% values < 45 % [33, 36]. Preoperative traction combined with respiratory exercise relieves the main curvature and improves pulmonary function by increasing the chest volume. A few studies have explored the pulmonary benefits afforded by traction, often in pediatric patients. Koller et al. reported an average improvement of 9 % among patients with preoperative FVC% values < 40 % [17]. Bogunovic et al. reported 9 % improvements in both the FEV1 % and FVC% in patients with severe, pediatric spinal deformities, thus more than we observed (5.15 and 6.76 %) [37]. Lenke et al. reported remarkable increases in the FEV1 % and FVC% (6.6 and 9.5 %) of patients with severe rigid kyphoscoliosis, similar to our results [27]. However, HGT is not strong, is inefficient, and cannot be used to treat severe rigid deformities. One prior study on the pulmonary function of patients with severe spinal deformities who underwent HGT reported no improvement in the PFT results [38].

We treat patients with extremely severe scoliosis (Cobb angle > 100°) using a staged strategy, thus initial preoperative HGT is followed by posterior pedicle screw-rod system internal fixation. In the process of traction, pulmonary function improves due to amelioration of the deformity and appropriate respiratory exercise. Most patients are satisfied with the therapeutic effects but some PFT results do not improve to an extent that allows patients to tolerate surgical correction. The traction effect of HGT can reach a plateau in a short time (within 2 weeks) due to its poor strength (up to one-half body weight) and low efficiency. Therefore, HPT has become the most commonly used traction technology for severe rigid scoliosis at present due to its powerful distraction forces. We found significantly more FVC% improvement in the HPT group than in the HGT group (6.76 ± 1.85 vs. 15.6 ± 3.47, *P* = 0.024). Although the HPT group exhibited somewhat more FEV% improvement than the HGT group, the difference was not significant (5.15 ± 2.27 vs. 11.76 ± 2.22 %, *P* = 0.91). Thus, HPT improved pulmonary function; this is a critical component of pre-surgery optimization.

However, some problems remain, principally complications associated with iliac puncture; this is associated with risks of damage to the main nerves or large blood vessels, causing paralysis or death [39, 40]. Stiffness and early cervical spine degeneration were evident in almost all patients of HPT [41]. In this study, neurological symptoms (cranial nerve symptoms, brachial plexus palsy, paresthesia and hypodynamia) were observed in 2 of the 30 patients of HPT, and cervical stiffness and early degeneration were observed in almost all patients of HPT in our study. Most of the patients had the pin tract infections, including exudation and even purulence, which need regular wound care. Also, HPT requires long-term hospitalization, reducing hospital income. Thus, HPT may not in practice be preferred to conventional preoperative traction.

There are some limitations in this study. First, this work limited by its retrospective nature and its small sample size, which requires further large sample and multicenter studies. Second, this study paid less attention to the power analysis, so the further long-term follow-up studies is necessary. Third, this study did not fully discuss the traction related complications, comorbidities and the corresponding management measures..

## Conclusions

In summary, we compared the efficacies of preoperative HGT and HPT in 81 patients with severe kyphoscoliosis and pulmonary insufficiency. Compared to the HGT group, patients who underwent preoperative HPT exhibited more satisfactory radiographic correction of their deformities, better pulmonary function, and a need for fewer osteotomies. HPT may be an useful preoperative technique for patients with extremely severe scoliosis and severe pulmonary dysfunction .

## Data Availability

The datasets used and/or analysed during the current study are available from the corresponding author on reasonable request.

## Abbreviations

HGT: Halo-gravity traction
HFT: Halo-femoral traction
HPT: Halo-pelvic traction
FVC%: Predicted forced vital capacity
FEV1%: Forced expiratory volume over 1s
